# Supplementary figures and images for: Differential p16 expression levels in the liver, hepatocytes and hepatocellular cell lines
Source: PeerJ. 2021 Nov 2;9:e12358. doi: 10.7717/peerj.12358 (PMC8570159; doi:10.7717/peerj.12358)

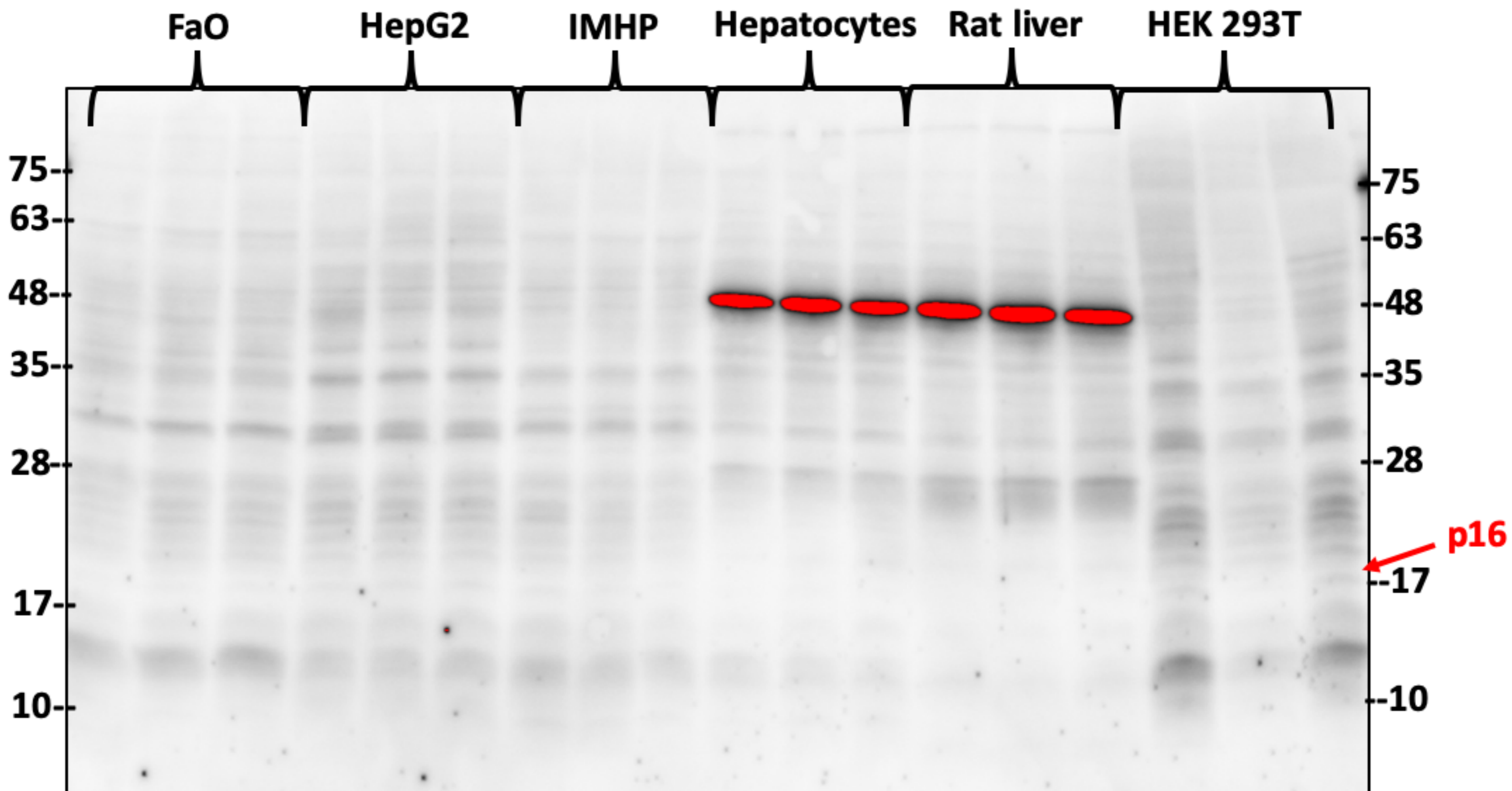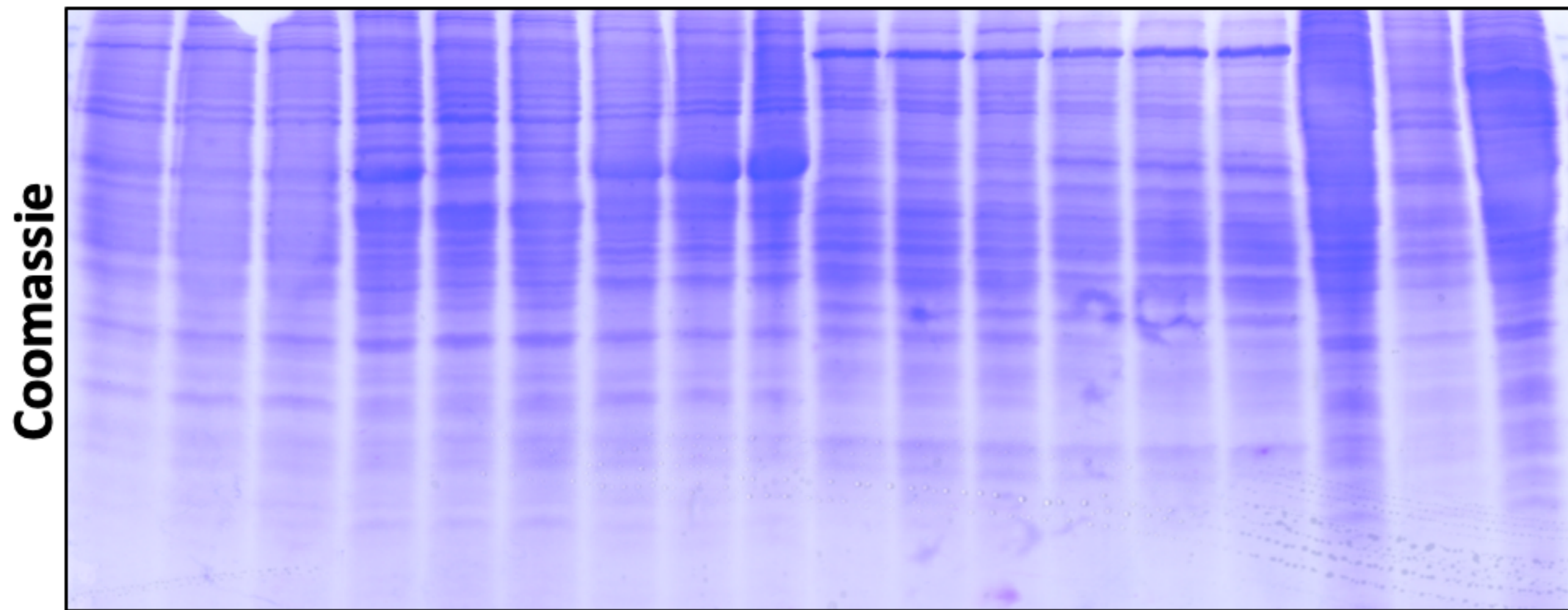

Supplement: Supplemental Information 1 — Immunoblot probed with a monoclonal antibody to p16. Each lane represents a lysate from one biological replicate. Underneath is a Coomassie-stained gel for loading control. [file peerj-09-12358-s001.pdf]
